# Controlled Synthesis and Infrared Emission Properties of Core–Shell TiO_2_ Hollow Microspheres

**DOI:** 10.3390/ma19071447

**Published:** 2026-04-04

**Authors:** Zeyu Liu, Yang Xiang, Zhihang Peng, Binzhi Jiang

**Affiliations:** 1 Science and Technology on Advanced Ceramic Fibers and Composites Laboratory, College of Aerospace Science and Engineering, National University of Defense Technology, Changsha 410073, China; 2 Institute of Mechanics, Chinese Academy of Sciences, Beijing 100190, China

**Keywords:** core–shell structure, TiO_2_, hollow microspheres, infrared emissivity, radiation cooling, solvothermal method

## Abstract

With the growing demand for advanced passive cooling technologies in fields such as building energy efficiency, thermal protection of electronic devices, and personal thermal comfort, radiative cooling materials have garnered considerable attention due to their ability to achieve cooling without external energy input. In this study, TiO_2_ hollow microspheres with a core–shell structure were successfully synthesized via a solvothermal method using TiCl_4_ as the titanium source and (NH_4_)_2_SO_4_ and CO(NH_2_)_2_ as structure-directing agents. The effects of reaction temperature (120–200 °C) and reaction time (0.5–36 h) on the morphology, crystal phase, specific surface area, pore structure, and infrared optical properties of the microspheres were systematically investigated. The results indicate that all prepared samples consisted of anatase-phase TiO_2_, with the microstructure significantly influenced by the synthesis conditions. An increase in reaction temperature promoted the transition from solid to hollow structures; the microspheres exhibited the most regular morphology and the largest specific surface area at 180 °C. Prolonging the reaction time facilitated the Ostwald ripening process, leading to a more complete hollow structure at 24 h. Infrared optical performance analysis revealed that all samples exhibited high emissivity approaching 100% in the 8–15 μm wavelength range, attributed to the intrinsic lattice vibration absorption of TiO_2_. In the 3–8 μm range, however, the emissivity was strongly modulated by the microstructure. Samples synthesized at 180 °C for 12–24 h demonstrated stable emissivity characteristics owing to their dense shells, uniform particle size, and well-defined hollow structures. This study elucidates the intrinsic relationship between microstructural evolution and infrared emission performance in TiO_2_ hollow microspheres, providing a theoretical foundation and process optimization strategy for their application in radiative cooling coatings, device thermal protection, and personal thermal management textiles.

## 1. Introduction

In the context of an increasingly pressing global energy crisis and climate change, the demand for advanced passive cooling technologies has become more urgent in critical areas such as building energy conservation, thermal protection of electronic devices, and personal thermal comfort [1]. Radiative cooling technology, as an innovative approach that enables cooling without external energy input, has attracted significant attention in recent years [2]. Its fundamental principle involves transferring heat directly through the atmospheric window to cold outer space via infrared radiation, thereby achieving a passive cooling effect [3]. The higher the infrared emissivity of a material, the stronger its radiative heat dissipation capability [4]. Therefore, developing materials with high emissivity in this spectral band is essential for enhancing radiative cooling performance. Titanium dioxide (TiO_2_), as a wide-bandgap semiconductor, has demonstrated significant potential for application in fields such as photocatalysis, photoelectric conversion, sensors, and optical coatings due to its excellent chemical and thermal stability, non-toxicity, and unique optical properties [5,6,7,8,9]. In particular, in the infrared region, the lattice vibration absorption of TiO_2_ imparts intrinsically high emissivity, providing it with a natural advantage for radiative heat dissipation in the mid-to-far-infrared band [10]. The common polymorphs of TiO_2_ include anatase, rutile, and brookite [11]. Due to their distinct crystal structures and corresponding phonon vibration modes, these polymorphs exhibit significantly different intrinsic infrared absorption characteristics [12,13]. Studies have shown that the trap depths of photogenerated electrons differ substantially among the three phases: anatase possesses the shallowest traps (<0.1 eV), rutile the deepest (approximately 0.9 eV), and brookite exhibits an intermediate value (around 0.4 eV) [14]. These differences in trap depth directly influence electron–hole recombination behavior and the probability of radiative recombination, leading to distinct mid-infrared emission properties across the different phases. This provides a theoretical foundation for tuning infrared emission performance through crystal phase engineering [15,16,17]. However, the high density and low specific surface area of solid TiO_2_ materials restrict their applicability in lightweight radiative cooling coatings or films. In recent years, TiO_2_ microspheres with hollow core–shell structures have attracted considerable attention due to their distinctive structural features. The hollow architecture not only significantly reduces material density [18] but also prolongs the propagation path of infrared radiation within the material, thereby enhancing the interaction between the radiation and the material and leading to excellent mid-infrared emission performance [19]. Moreover, the high specific surface area and well-developed pore structure facilitate efficient solar light scattering and provide ample space for surface functionalization [20]. Additionally, the hollow configuration effectively suppresses heat conduction, endowing the material with low thermal conductivity and further improving its thermal management capability [21]. Consequently, hollow core–shell structured TiO_2_ microspheres exhibit unique advantages in balancing high solar reflectance, high infrared emissivity, and low thermal conductivity, positioning them as ideal functional units for the development of advanced radiative cooling coatings.

At present, there are numerous reports in the literature on the synthesis, characterization, and application of TiO_2_ microspheres. In terms of preparation strategies, the template method is widely adopted due to its strong structural controllability. For example, Li et al. used chitosan/gelatin microspheres as templates, deposited TiO_2_ via the sol–gel method, and removed the templates by high-temperature sintering to successfully prepare TiO_2_ hollow microspheres and investigate their in vitro biocompatibility as cell carriers [22]. Ferreira et al. employed SiO_2_ microspheres as templates to prepare Ag-doped TiO_2_ hollow microspheres via the sol–gel method and combined this with molecular imprinting technology to achieve the selective photodegradation of bilirubin [23]. Although significant progress has been made in the preparation and application of TiO_2_ hollow microspheres in the aforementioned studies, certain limitations still exist regarding the preparation methods. While the traditional template method can effectively construct hollow structures, it often involves multi-step operations and template removal processes, which complicate the procedure and may introduce impurities. In contrast, the solvothermal method, a widely adopted synthesis strategy in recent years, enables the one-step formation of hollow core–shell structures without the need for additional templates, offering advantages such as a simple process, strong controllability, and high product purity. However, existing research has mostly focused on the isolated effects of solvothermal parameters on the morphology or crystalline phase of TiO_2_ hollow microspheres, or has primarily addressed their performance in fields such as photocatalysis and energy storage. However, systematic studies on the intrinsic relationships among solvothermal preparation conditions, microstructure, crystal phase composition, and infrared emission performance of TiO_2_ hollow microspheres remain rarely reported.

In this study, core–shell structured TiO_2_ hollow microspheres were synthesized via a solvothermal method using TiCl_4_ as the titanium source and (NH_4_)_2_SO_4_ and CO(NH_2_)_2_ as structure-directing agents. The effects of reaction temperature and time on the morphology, crystal structure, specific surface area, pore structure, and infrared optical properties of the microspheres were systematically investigated. By elucidating the intrinsic relationship between microstructural evolution and infrared emissivity, this work aims to provide a theoretical foundation and process optimization pathway for the application of hollow-structured TiO_2_ materials in radiative cooling and thermal management technologies.

## 2. Experimental

### 2.1. Materials

Titanium tetrachloride (TiCl_4_, analytical grade, China National Pharmaceutical Group Chemical Reagent Co., Ltd., Shanghai, China), ammonium sulfate ((NH_4_)_2_SO_4_, analytical grade, China National Pharmaceutical Group Chemical Reagent Co., Ltd.), urea (CO(NH_2_)_2_, analytical grade, China National Pharmaceutical Group Chemical Reagent Co., Ltd.), anhydrous ethanol (analytical grade, Chengdu Cologne Chemical Co., Ltd., Chengdu, China), and deionized water (prepared in the laboratory).

### 2.2. Fabrication of Core–Shell Structured TiO_2_ Microspheres

**Figure 1:**
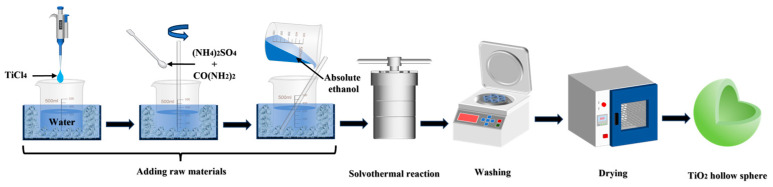
Fabrication process of TiO_2_ hollow microspheres.

Figure 1 shows the schematic procedure for the preparation of core–shell TiO_2_ hollow microspheres. Under ice-bath conditions, 5 mL of TiCl_4_ was added dropwise to 60 mL of deionized water under continuous stirring to control the hydrolysis rate, yielding an aqueous TiCl_4_ solution. Subsequently, 2 g of (NH_4_)_2_SO_4_ and 16 g of CO(NH_2_)_2_ were sequentially added and stirred until completely dissolved. While maintaining stirring, 30 mL of ethanol was slowly introduced dropwise to form a homogeneous precursor solution, at which point the total volume of the solution was approximately 95 mL. The resulting mixture was transferred into a 200 mL autoclave and subjected to a solvothermal reaction at a preset temperature. After the reaction was completed, the precipitate was collected and washed repeatedly with ethanol and deionized water. Following drying, core–shell TiO_2_ microspheres were obtained. By varying the reaction temperature and time, the effects of different synthesis conditions on the microstructure and properties of the samples were systematically investigated.

### 2.3. Sample Characterization

The microstructure of the core–shell TiO_2_ hollow microspheres was characterized using a TESCAN CLARA scanning electron microscope (SEM) (TESCAN, Brno, Czech Republic) and a JEOL JEM-F200 transmission electron microscope (TEM) (JEOL, Tokyo, Japan). Particle size distribution was determined with a Bettersize 2600 laser particle size analyzer (Bettersize Instruments Ltd., Dandong, China). Specific surface area and pore structure were analyzed using a Quantachrome Autosorb iQ gas adsorption analyzer (Quantachrome Instruments, Grafton, MA, USA). Prior to measurement, the samples were degassed at 250 °C, and nitrogen adsorption–desorption isotherms were collected at 77 K. The specific surface area was calculated using the Brunauer–Emmett–Teller (BET) method. The phase composition of the samples synthesized under different conditions was examined by X-ray diffraction (XRD) using a Malvern Panalytical X’Pert^3^ Powder diffractometer (Malvern Panalytical, Malvern, UK) over a 2θ range from 5° to 90°. Infrared spectra in the 4000–400 cm^−1^ region were recorded using a Shimadzu IRTracer-100 Fourier transform infrared spectrometer (FTIR) (Shimadzu, Kyoto, Japan). Mid-infrared reflectance measurements were performed using a Nicolet iS50 Fourier transform infrared spectrometer (Thermo Fisher Scientific, Waltham, MA, USA) equipped with an integrating sphere and a gold mirror reflection accessory. Spectra were collected over the range of 2.5–15 μm using a gold substrate as the background reference, and the emissivity was subsequently calculated from the reflectance data.

## 3. Results and Discussion

### 3.1. Formation Mechanism of Core–Shell TiO_2_ Hollow Microspheres

The formation of core–shell structured TiO_2_ hollow microspheres is primarily attributed to the synergistic effect of the Ostwald ripening mechanism and structure-directing agents. In the initial stage of the solvothermal reaction, TiO_2_ nanoparticles generated by TiCl_4_ hydrolysis aggregate to form solid microsphere precursors. As the reaction proceeds, urea decomposes and releases OH^−^, creating a weakly alkaline environment. The in situ generated gas microbubbles serve as a soft template, inducing the formation of a loose interior structure and resulting in a unique configuration with a loose core and relatively dense shell. This density gradient provides the driving force for the subsequent Ostwald ripening process [24,25]. Meanwhile, SO_4_^2−^ ions released from the decomposition of (NH_4_)_2_SO_4_ play a crucial structure-directing role. Due to their high coordination affinity, SO_4_^2−^ ions preferentially adsorb onto the particle surfaces, inhibiting excessive grain growth and stabilizing the outer shell, thereby promoting the formation of hollow structures [26,27,28]. The addition of ethanol further regulates the nucleation and growth behavior of the precursors. On the one hand, as an organic solvent, ethanol effectively reduces the water activity in the system, slowing down the hydrolysis rate of TiCl_4_ and ensuring more uniform and controllable nucleation. On the other hand, the coordination effect of ethanol facilitates the formation of stable precursor species, promoting the generation of uniform solid sphere precursors that are essential for the subsequent inside-out Ostwald ripening [29]. Under the synergistic influence of the aforementioned factors, nanoparticles with higher surface energy in the interior of the microspheres gradually dissolve. The dissolved species diffuse outward and re-deposit onto the microsphere surface. This internal dissolution–external deposition process progressively hollows out the microspheres, ultimately yielding a core–shell structure with a well-defined shell [30]. Further optimization of the reaction temperature and time promotes the rearrangement and densification of the nanoparticles, resulting in more regular hollow architectures. Consequently, core–shell TiO_2_ hollow microspheres with high specific surface area, uniform particle size, and intact shell layers are obtained.

### 3.2. Microstructural and Phase Characterization of Core–Shell TiO_2_ Hollow Microspheres

The solvothermal reaction temperature and time play a decisive role in the microstructural evolution and infrared emission properties of core–shell TiO_2_ hollow microspheres [31]. To elucidate their regulatory effects, this section systematically investigates the influence of different reaction temperatures (120–200 °C) and reaction times (0.5–36 h) on the structure and properties of the samples. The analysis focuses on four key aspects: morphological evolution, particle size distribution and specific surface area, crystal phase and chemical composition, and infrared emission performance, aiming to establish the intrinsic correlations between these parameters and the material’s functionality.

#### 3.2.1. Morphological Evolution

**Figure 2:**
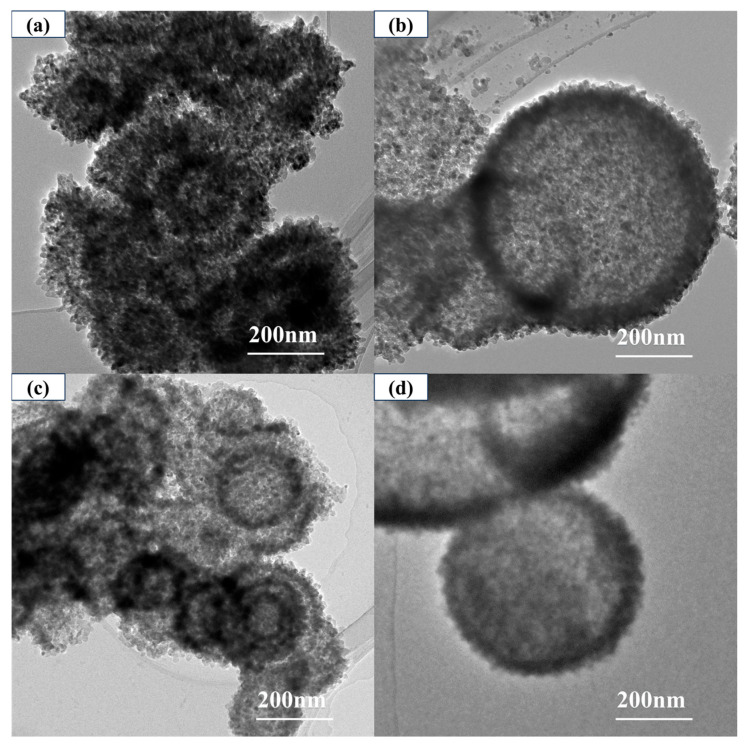
TEM images of the TiO_2_ hollow microspheres synthesized at various temperatures: (**a**) 120 °C, (**b**) 160 °C, (**c**) 180 °C, and (**d**) 200 °C.

Figure 2 shows the TEM images of core–shell TiO_2_ hollow microspheres prepared at different reaction temperatures. At a reaction temperature of 120 °C, the samples had already formed microspherical morphology, but their interiors remained solid. As the temperature increased to 160 °C, the microspheres began to exhibit distinct hollow structures, with wall thicknesses ranging from approximately 40–100 nm.

**Figure 3:**
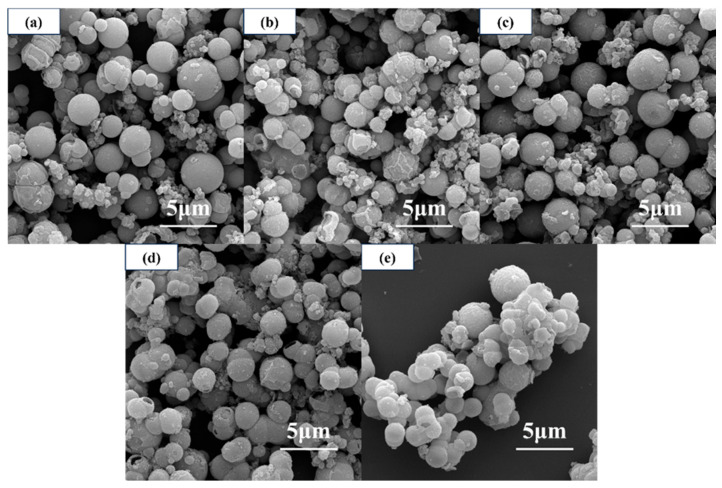
SEM images of core–shell structured TiO_2_ microspheres prepared at different reaction temperatures: (**a**) 120 °C, (**b**) 140 °C, (**c**) 160 °C, (**d**) 180 °C, and (**e**) 200 °C.

Figure 3 shows the SEM images of core–shell structured TiO_2_ microspheres prepared at different reaction temperatures. As can be seen, the reaction temperature significantly influences the microsphere morphology. At lower temperatures (≤140 °C), the microspheres exhibited a rough, textured surface with an uneven particle size distribution. As the temperature increased, the surface gradually became smoother and the particle size distribution more uniform, with surface roughness reaching a minimum at 180 °C. However, when the temperature was further increased to 200 °C, noticeable adhesion between microspheres occurred, and their dispersibility began to decline.

**Figure 4:**
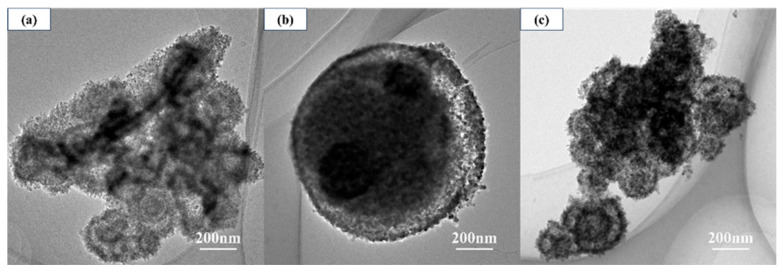
TEM images of TiO_2_ hollow microspheres prepared at different reaction times: (**a**) 0.5 h, (**b**) 6 h, and (**c**) 24 h.

Figure 4 shows the TEM images of core–shell TiO_2_ hollow microspheres prepared at different reaction times. At a reaction time of 0.5 h, microsphere structures had already emerged; however, the presence of numerous surrounding nanoparticles obscured the observation of complete microsphere morphology, and hollow interiors had not yet formed. When the reaction time was extended to 6 h, relatively complete microsphere structures were observed, but the hollow architecture remained poorly developed, with only incipient hollow regions appearing near the shell edge. Upon further prolonging the reaction time to 24 h, the hollowness of the microspheres was significantly enhanced, ultimately yielding TiO_2_ microspheres with well-defined hollow structures. Overall, as the reaction time increased, the hollow structure of the TiO_2_ microspheres gradually evolved and became progressively perfected.

**Figure 5:**
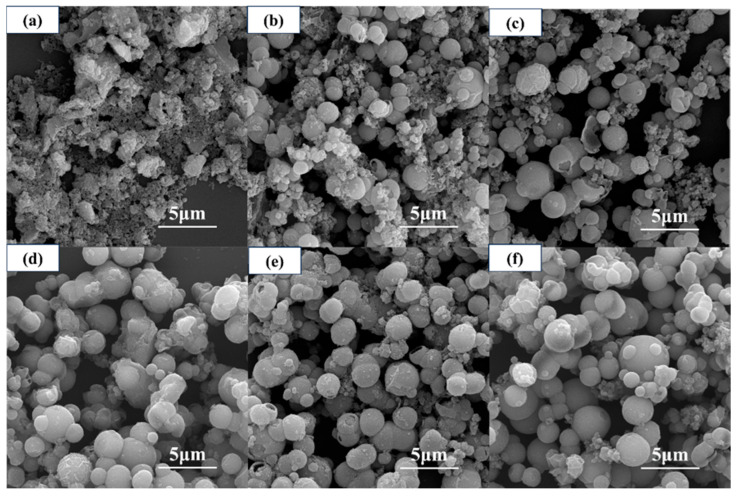
SEM images of TiO_2_ hollow microspheres prepared at different reaction times: (**a**) 0.5 h, (**b**) 3 h, (**c**) 6 h, (**d**) 12 h, (**e**) 24 h, and (**f**) 36 h.

Figure 5 shows the SEM images of core–shell TiO_2_ hollow microspheres prepared at different reaction times. At a reaction time of 0.5 h, the sample surface was covered with numerous amorphous nanoparticles, making it difficult to discern complete microsphere structures. When the reaction time was extended to 3–6 h, microsphere structures began to form; however, some microspheres exhibited relatively rough surfaces and remained surrounded by abundant amorphous nanoparticles. As the reaction time was further prolonged, these nanoparticles gradually diminished. At 12 h, the nanoparticles around the microspheres had largely disappeared, although some microsphere surfaces still appeared slightly rough. Further extending the reaction time to 24 or 36 h resulted in progressively smoother microsphere surfaces.

In summary, optimizing both the reaction temperature and time is essential for obtaining hollow microspheres with regular morphology and intact shells. An appropriate temperature (180 °C) promotes the formation of smooth surfaces and well-defined hollow structures, whereas an excessively high temperature (200 °C) leads to microsphere adhesion. Similarly, extending the reaction time to 24 h or longer further perfects the hollow architecture. Thus, 180 °C and a reaction time of at least 24 h serve as key control points for achieving optimal morphological evolution.

#### 3.2.2. Particle Size Distribution and Specific Surface Area

Variations in reaction temperature and time not only influence the morphology of the microspheres but also significantly govern their particle size distribution, specific surface area, and pore structure—parameters that are closely linked to the material’s optical properties and adsorption capacity.

**Figure 6:**
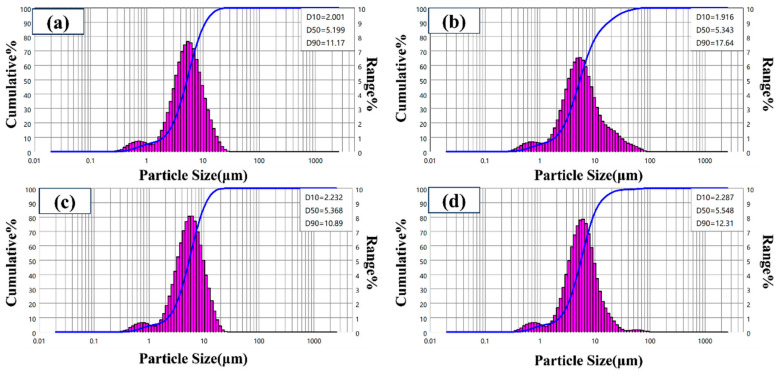
Particle size distribution of TiO_2_ hollow microspheres prepared at different reaction temperatures: (**a**) 120 °C, (**b**) 140 °C, (**c**) 180 °C, and (**d**) 200 °C.

Figure 6 shows the particle size distribution of core–shell TiO_2_ hollow microspheres prepared at different reaction temperatures. The microspheres exhibited an average size distribution ranging from 0.5 to 20 μm, with the particle size gradually increasing as the reaction temperature rose. Notably, all particle size distribution curves displayed a small peak in the range of 0.5–1 μm, indicating a bimodal distribution. TEM analysis (Figure 2) confirmed that these small particles also possessed a hollow structure; therefore, they are not incompletely assembled primary TiO_2_ nanoparticles but rather independently formed small-sized hollow microspheres. During the solvothermal process, variations in local titanium source concentration, nucleation time, or reaction driving force lead to the coexistence of hollow microspheres with different sizes: large microspheres corresponding to the main peak (several to tens of micrometers) and small microspheres corresponding to the minor peak (submicron scale). Both types share the same formation mechanism, following the Ostwald ripening process described in Section 3.1—the initial formation of solid precursors, followed by internal dissolution and external deposition, ultimately yielding hollow structures. At a reaction temperature of 180 °C, the main peak shifts toward larger particle sizes and exhibits the narrowest distribution, indicating that the assembly of large microspheres is more complete and that the size uniformity is optimal at this temperature, although a small population of small microspheres still persists.

Table 1 summarizes the specific surface area, average pore volume, and average pore size of core–shell TiO_2_ hollow microspheres prepared at different reaction temperatures. As the reaction temperature increased, the specific surface area and pore volume first increased and then decreased, while the average pore size exhibited the opposite trend, initially decreasing followed by an increase. At lower temperatures, the driving force for the reaction is insufficient, hindering the effective assembly of nanoparticles into hollow structures and resulting in the formation of loosely packed solid microspheres. The low degree of hollowness contributes to relatively small specific surface area and pore volume, while the loose packing leads to larger interparticle gaps, accounting for the larger average pore size. With increasing temperature, the enhanced reaction driving force facilitates the directional rearrangement and densification of nanoparticles, promoting the formation of well-defined, compact hollow microspheres. This structural evolution leads to a marked increase in specific surface area and pore volume, both reaching optimal values at 180 °C. However, when the temperature is further raised to 200 °C, excessive nanoparticle growth occurs, resulting in grain coarsening, shell densification, and even local sintering, which in turn reduces the specific surface area and pore volume. The variation in average pore size reflects the evolution of intergranular pore structures. Within an appropriate temperature range (e.g., 160 °C), sufficient nanoparticle rearrangement and uniform grain size generate relatively small intergranular pores, minimizing the pore size. As the temperature continues to rise, grain growth and widening of grain boundary gaps lead to a progressive increase in average pore size.

**Figure 7:**
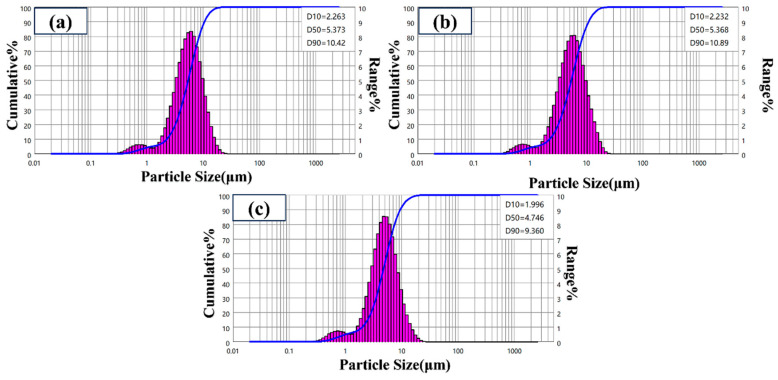
Particle size distribution of TiO_2_ hollow microspheres prepared at different reaction times: (**a**) 12 h, (**b**) 24 h, and (**c**) 36 h.

Figure 7 shows the particle size distribution of core–shell TiO_2_ hollow microspheres prepared at different reaction times. The results indicate that the particle sizes of samples obtained at 12 h and 24 h are essentially similar. However, when the reaction time was extended to 36 h, the particle size began to exhibit a decreasing trend. This phenomenon may be attributed to two factors: first, in the later stage of the reaction, the pre-formed hollow structure may undergo shell densification and contraction to minimize surface energy; second, prolonged exposure to the solvothermal and alkaline environment may induce chemical etching on the microsphere surface, leading to shell thinning. Additionally, all samples prepared under various time conditions exhibited a small peak in the range of 0.5–1 μm, revealing a bimodal distribution characteristic. TEM analysis confirmed that these small-sized particles also possess a hollow structure, consistent with the formation mechanism of larger hollow microspheres. Their presence is attributed to differences in local nucleation and growth conditions during the reaction process.

Table 2 summarizes the specific surface area, average pore volume, and average pore size of core–shell TiO_2_ hollow microspheres prepared at different reaction times. As the reaction time increased, the specific surface area exhibited a continuous increasing trend: it remained relatively low at 0.5 h and 3 h, then increased sharply when the reaction time was extended to 6 h, reaching its maximum value at 24 h. The average pore volume first increased and then slightly decreased with prolonged reaction time, peaking at 6 h. The average pore size was relatively large in the early stage of the reaction (≤3 h), decreased sharply after 6 h, and subsequently stabilized. These phenomena can be attributed to the following mechanism. In the early stage of the solvothermal reaction (≤3 h), TiO_2_ nanoparticles generated by TiCl_4_ hydrolysis gradually aggregate to form relatively dense microsphere precursors, resulting in a low specific surface area and larger interparticle pores, which account for the larger average pore size. As the reaction progresses to the intermediate stage (around 6 h), under the action of OH^−^ ions released by urea decomposition, a dissolution–redeposition process (Ostwald ripening) occurs within the microspheres, gradually forming a hollow structure. Simultaneously, SO_4_^2−^ ions released from (NH_4_)_2_SO_4_ adsorb onto the TiO_2_ surface, inhibiting grain growth and causing the shell to consist of ultrafine nanocrystals. This leads to a sharp increase in specific surface area and a rapid decrease in average pore size. During this process, nanoparticle rearrangement increases interparticle spacing, resulting in the maximum average pore volume. In the later stage of the reaction (6–24 h), the hollow structure becomes further developed, the shell nanocrystals continue to refine, the specific surface area further increases, and the pore size stabilizes within the microporous range. Meanwhile, as the microsphere structure becomes denser, some large pores disappear or transform into micropores, leading to a slight decrease in average pore volume.

A comparison of the two variables revealed that 180 °C and 24 h are the critical conditions for achieving the maximum specific surface area and optimal pore structure, respectively. Excessive temperature (200 °C) leads to grain coarsening and a reduction in specific surface area, while prolonged reaction time (36 h) results in structural degradation and decreased particle size. This indicates that a judicious combination of temperature and time is essential for obtaining high specific surface area and uniform pore size.

#### 3.2.3. Crystal Phase and Chemical Composition

The crystal phase and chemical composition of a material are key determinants of its intrinsic physical and chemical properties. In this section, the effects of different reaction temperatures and times on the crystal structure and chemical composition of core–shell TiO_2_ hollow microspheres are investigated through XRD and FTIR analysis.

**Figure 8:**
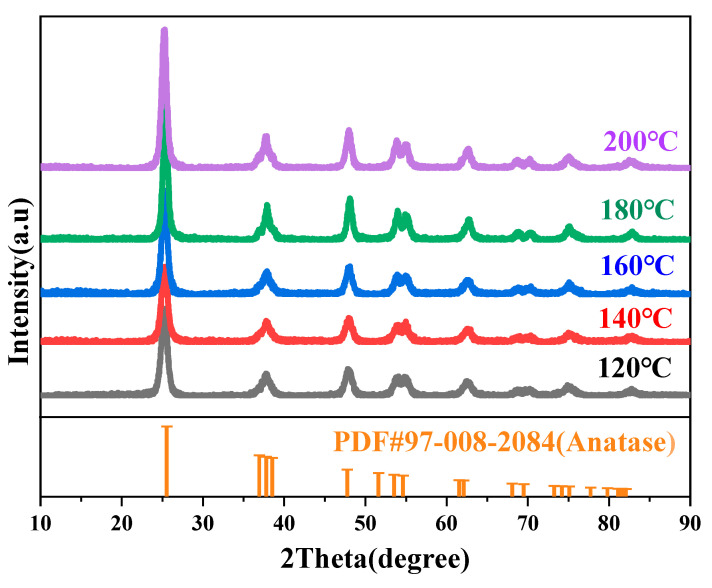
XRD patterns of TiO_2_ hollow microspheres prepared at different reaction temperatures.

Figure 8 shows the XRD patterns of core–shell TiO_2_ hollow microspheres prepared at different reaction temperatures. All samples exhibited diffraction peaks at 25.2°, 37.6°, 47.8°, 53.6°, 61.8°, 69.9°, 74.7°, 78.3°, and 82.3°. These peak positions are consistent with the standard characteristic peaks of anatase TiO_2_ [32], indicating that the as-prepared samples possess an anatase structure. This result also demonstrates that variations in reaction temperature do not alter the crystal phase of the core–shell TiO_2_ hollow microspheres.

**Figure 9:**
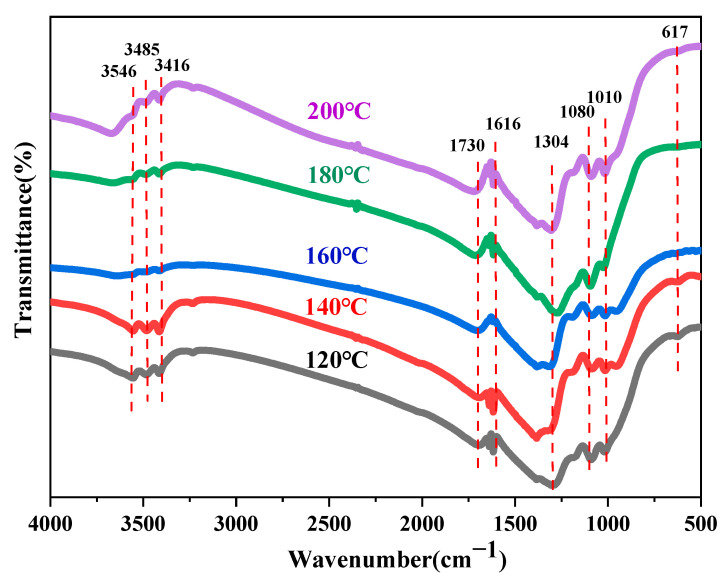
FTIR spectra of TiO_2_ hollow microspheres prepared at different reaction temperatures.

Figure 9 shows the FTIR spectra of core–shell TiO_2_ hollow microspheres prepared at different reaction temperatures. The FTIR curves of samples obtained at various temperatures exhibited no significant differences, indicating that their chemical compositions are essentially identical. The absorption band in the region around 3546 cm^−1^ corresponds to O–H stretching vibrations, suggesting the presence of trace amounts of residual water or ethanol on the sample surface [33]. The bands at 3485 cm^−1^ and 3416 cm^−1^ are attributed to N–H stretching vibrations [34,35], likely originating from residual (NH_4_)_2_SO_4_ and CO(NH_2_)_2_. The peak at 1730 cm^−1^ may be assigned to C=O stretching vibrations [36], possibly derived from residual CO(NH_2_)_2_. The band at 1616 cm^−1^ is attributable to N–H bending vibrations [37], which may also arise from residual (NH_4_)_2_SO_4_ and CO(NH_2_)_2_. The transmission peak at 1304 cm^−1^ corresponds to C–N stretching vibrations [38], likely originating from residual CO(NH_2_)_2_. The bands at 1080 cm^−1^ and 1010 cm^−1^ are associated with S=O stretching vibrations and C–O stretching vibrations, respectively [39,40], which may originate from (NH_4_)_2_SO_4_ and ethanol. Additionally, the absorption peak at 617 cm^−1^ corresponds to the characteristic vibration of TiO_2_ [41].

**Figure 10:**
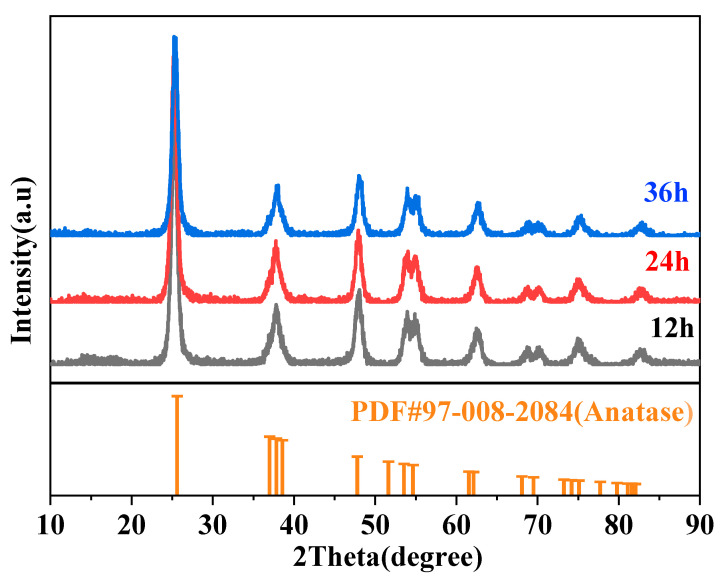
XRD patterns of TiO_2_ hollow microspheres prepared at different reaction times.

Figure 10 shows the XRD patterns of core–shell TiO_2_ hollow microspheres prepared at different reaction times. All samples exhibited diffraction peaks at 25.2°, 37.6°, 47.8°, 53.6°, 61.8°, 69.9°, 74.7°, 78.3°, and 82.3°. These peak positions are consistent with the standard characteristic peaks of anatase TiO_2_ [32], indicating that the as-prepared samples possess an anatase structure. This result also demonstrates that variations in reaction time do not alter the crystal phase of the core–shell TiO_2_ hollow microspheres.

**Figure 11:**
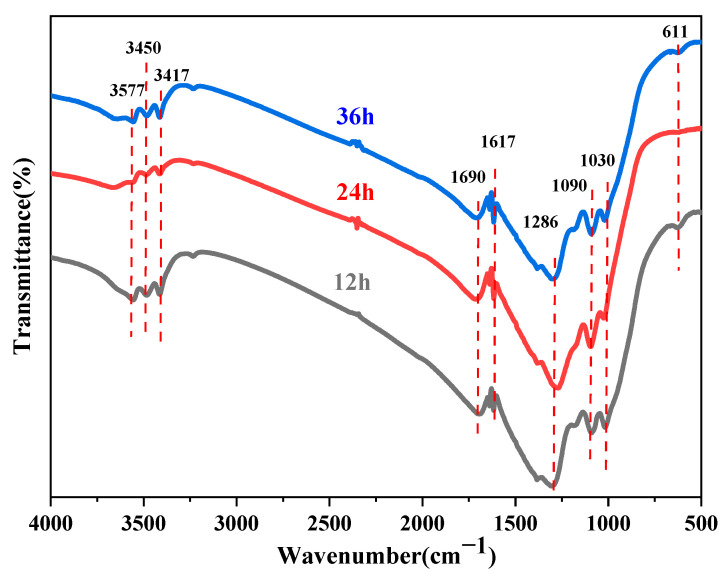
FTIR spectra of TiO_2_ hollow microspheres prepared at different reaction times.

Figure 11 shows the FTIR spectra of core–shell TiO_2_ hollow microspheres prepared at different reaction times. The spectra of samples obtained under various reaction times exhibited no significant differences, indicating that their chemical compositions are essentially identical. The absorption band around 3577 cm^−1^ corresponds to O–H stretching vibrations [33], suggesting the presence of trace amounts of residual water or ethanol on the sample surface. The broad band in the range of 3450–3417 cm^−1^ is attributed to N–H stretching vibrations [35], likely originating from residual (NH_4_)_2_SO_4_ and CO(NH_2_)_2_. The peak at 1690 cm^−1^ may be assigned to C=O stretching vibrations [42], possibly derived from residual CO(NH_2_)_2_. The band at 1617 cm^−1^ corresponds to N–H bending vibrations [37], which may also arise from residual (NH_4_)_2_SO_4_ and CO(NH_2_)_2_. The peak at 1286 cm^−1^ is attributable to C–N stretching vibrations [43], likely originating from residual CO(NH_2_)_2_. The broad band in the range of 1090–1030 cm^−1^ is associated with S=O stretching vibrations and C–O stretching vibrations [44,45], which may originate from (NH_4_)_2_SO_4_ and ethanol, respectively. In addition, the absorption peak at 611 cm^−1^ corresponds to the characteristic vibration of TiO_2_ [46].

### 3.3. Infrared Emission Properties of Core–Shell TiO_2_ Hollow Microspheres

**Figure 12:**
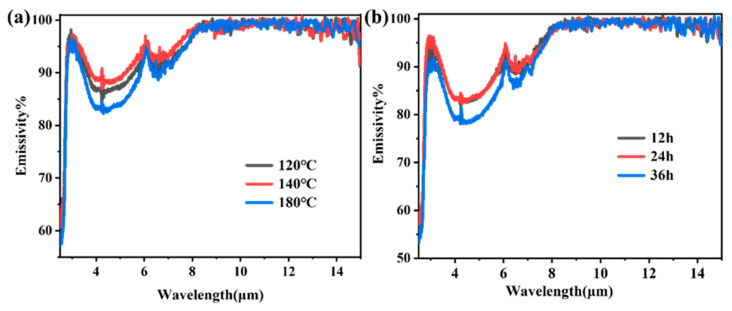
Infrared emissivity of core–shell TiO_2_ hollow microspheres prepared under different conditions: (**a**) reaction temperature; (**b**) reaction time.

Figure 12 illustrates the infrared emissivity spectra of samples prepared under different reaction conditions. As shown, the emissivity of all samples was approximately 55% near 2.5 μm, followed by a sharp increase to nearly 100% around 3 μm. Within the atmospheric window region (8–15 μm), all samples exhibited emissivity values close to 100%, which were notably higher than that of conventional rutile TiO_2_ powder (~90%) [47] and other commonly used oxide ceramics [48]. This behavior can be attributed to the strong intrinsic absorption arising from TiO_2_ lattice vibrations in the long-wavelength range combined with the influence of the unique core–shell hollow microstructure [49]. These findings suggest that core–shell structured TiO_2_ hollow microspheres inherently possess superior emissivity characteristics in the critical radiative cooling band. In the 3–8 μm wavelength range, the emissivity is modulated by microstructural features and exhibits a “limited but discernible” variation pattern. From the perspective of reaction temperature (120–180 °C), the emissivity follows the order: 140 °C > 120 °C > 180 °C. The sample prepared at 180 °C, characterized by a dense and intact shell along with the narrowest particle size distribution, effectively suppressed infrared absorption within the microspheres, thus exhibiting the lowest emissivity. In contrast, the 140 °C sample, with its rough surface and loose structure, displayed the strongest scattering effects and consequently the highest emissivity. Regarding reaction time, the emissivity followed the trend: 36 h > 12 h ≈ 24 h. At intermediate reaction times (12–24 h), the microspheres developed a well-defined hollow structure with a dense shell and uniform particle size, which minimizes light absorption and results in low and stable emissivity. However, when the reaction time was extended to 36 h, excessive maturation leads to microstructural degradation—including reduced particle size, increased defect density, and localized fragmentation—which enhances multiple scattering and light trapping, thereby significantly increasing emissivity. It is worth noting that the variation in emissivity across the entire 3–8 μm band remained relatively modest (ranging from approximately 0.7 to 0.9), and the emissivity in the long-wavelength region (>8 μm) was virtually unaffected by synthesis conditions. This behavior arises from the dual dependence of TiO_2_ infrared emission on both intrinsic absorption and microstructure. In the long-wavelength band, the strong intrinsic absorption of TiO_2_ lattice vibrations predominated, masking any microstructural influence and yielding a consistently high emissivity near 100% for all samples. This represents a key advantage of TiO_2_ as a radiative cooling material: high atmospheric window emissivity can be achieved without complex structural engineering. In the mid-wavelength band, although intrinsic absorption remained significant, microstructural features such as shell density, particle size uniformity, and hollow integrity exerted a distinguishable modulation on light scattering and absorption efficiency. Although the modulation amplitude was limited (approximately 0.2), this “intrinsically dominated, structurally fine-tuned” characteristic holds practical significance, enabling TiO_2_ hollow microspheres to fulfill fundamental radiative cooling functions while allowing performance customization for specific application requirements through process optimization.

In summary, optimizing reaction temperature and time effectively tailors the microstructure of core–shell TiO_2_ hollow microspheres, thereby modulating their infrared emission performance in the 3–8 μm band. This provides a viable strategy for enhancing the performance of radiative cooling materials.

### 3.4. Application Prospects

**Figure 13:**
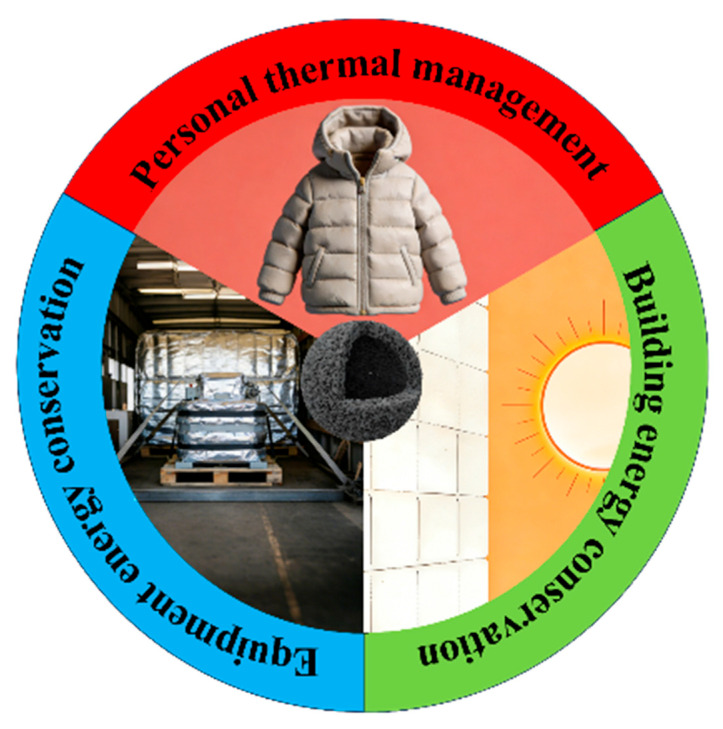
Application prospects of core–shell structured TiO_2_ hollow microspheres.

Based on the core–shell TiO_2_ hollow microspheres prepared in this study, their high infrared emissivity and unique microstructural features endow them with broad application prospects in several key fields, as illustrated in Figure 13.

In the field of building energy conservation, these TiO_2_ hollow microspheres can be incorporated as functional fillers into coating matrices to fabricate radiative cooling exterior wall coatings or roof coverings with efficient passive cooling capabilities. Their unique hollow architecture significantly enhances the solar light scattering efficiency, thereby reducing the surface absorption of solar radiation. Simultaneously, the intrinsic lattice vibrations of TiO_2_ enable high infrared emissivity within the 8–13 μm atmospheric window, facilitating continuous heat dissipation into outer space. This passive cooling strategy, requiring no external energy input, offers an effective means to reduce building air conditioning loads during summer and mitigate the urban heat island effect. Moreover, given the well-established thermal insulation advantages of hollow microsphere materials reported in the literature [50,51,52], the structural features of the present TiO_2_ hollow microspheres suggest promising potential for achieving multifunctional integration of radiative cooling and thermal insulation in building energy conservation applications.

In the field of equipment energy conservation, this material can be applied to the surfaces of outdoor communication base stations, power transmission facilities, storage tanks, and data centers that require stringent thermal management [53,54,55]. Prolonged exposure of outdoor equipment to solar radiation often leads to increased internal temperatures, reduced operational efficiency, and even malfunction or failure. Incorporating TiO_2_ hollow microspheres into heat dissipation coating systems can reduce the surface heat load of equipment through their high solar reflectance while simultaneously dissipating accumulated heat via mid-infrared radiation, thereby enabling passive thermal management.

In the field of personal thermal management, TiO_2_ hollow microspheres can be integrated with textile fibers or coated onto fabric surfaces to develop smart textiles with radiative cooling functionality [56,57,58]. Owing to their high infrared emissivity, the TiO_2_ hollow microspheres prepared in this study can dissipate surface heat from the human body or fabric to the external environment via infrared radiation through the atmospheric window, achieving passive personal cooling. This approach offers a promising strategy for ensuring human thermal comfort in complex thermal environments.

## 4. Conclusions

This study systematically investigates the microstructural evolution and infrared emission properties of core–shell TiO_2_ hollow microspheres synthesized under solvothermal conditions. The results reveal that reaction temperature is the key factor determining the transition from solid to hollow structures, with 180 °C identified as the optimal temperature, yielding the most regular morphology and the largest specific surface area. Reaction time governs the hollowing process through Ostwald ripening, with 24 h being the optimal duration for achieving a well-defined hollow architecture. All samples exhibited intrinsic high emissivity approaching 100% in the 8–15 μm atmospheric window. More importantly, this study demonstrates that emissivity in the 3–8 μm range can be effectively modulated by tailoring the microstructure. This work not only elucidates the intrinsic structure–property relationship of TiO_2_ hollow microspheres but also confirms that materials with regular morphology, high specific surface area, and stable infrared emission performance can be obtained through optimized solvothermal parameters. These findings provide a theoretical foundation and process optimization pathway for the development of high-performance radiative cooling functional units based on TiO_2_ hollow microspheres, with promising applications in energy-efficient buildings, thermal protection of outdoor equipment, and personal thermal management textiles.

## Figures and Tables

**Table 1:** Specific surface area, average pore volume, and average pore size of TiO_2_ hollow microspheres prepared at different reaction temperatures.

Temperature (°C)	S_BET_ (m^2^/g)	Pore Volume (cm^3^/g)	Pore Diameter (nm)
120	168.923	0.140	0.793
160	305.045	0.184	0.604
180	381.871	0.267	0.635
200	238.942	0.196	0.708

**Table 2:** Specific surface area, average pore volume, and average pore size of TiO_2_ hollow microspheres prepared at different reaction times.

Time (h)	S_BET_ (m^2^/g)	Pore Volume (cm^3^/g)	Pore Diameter (nm)
0.5	32.375	0.171	17.457
3	54.816	0.265	12.458
6	239.553	0.313	0.651
24	381.871	0.267	0.635

## Data Availability

The original contributions presented in this study are included in the article. Further inquiries can be directed to the corresponding authors.
